# Supplementary material for: Interactions of Different Streptomyces Species and Myxococcus xanthus Affect Myxococcus Development and Induce the Production of DK-Xanthenes
Source: Int J Mol Sci. 2023 Oct 27;24(21):15659. doi: 10.3390/ijms242115659 (PMC10649082; doi:10.3390/ijms242115659)
Supplement: Supplementary file 1 [file ijms-24-15659-s001.zip › ijms-2646862-supplementary.pdf]

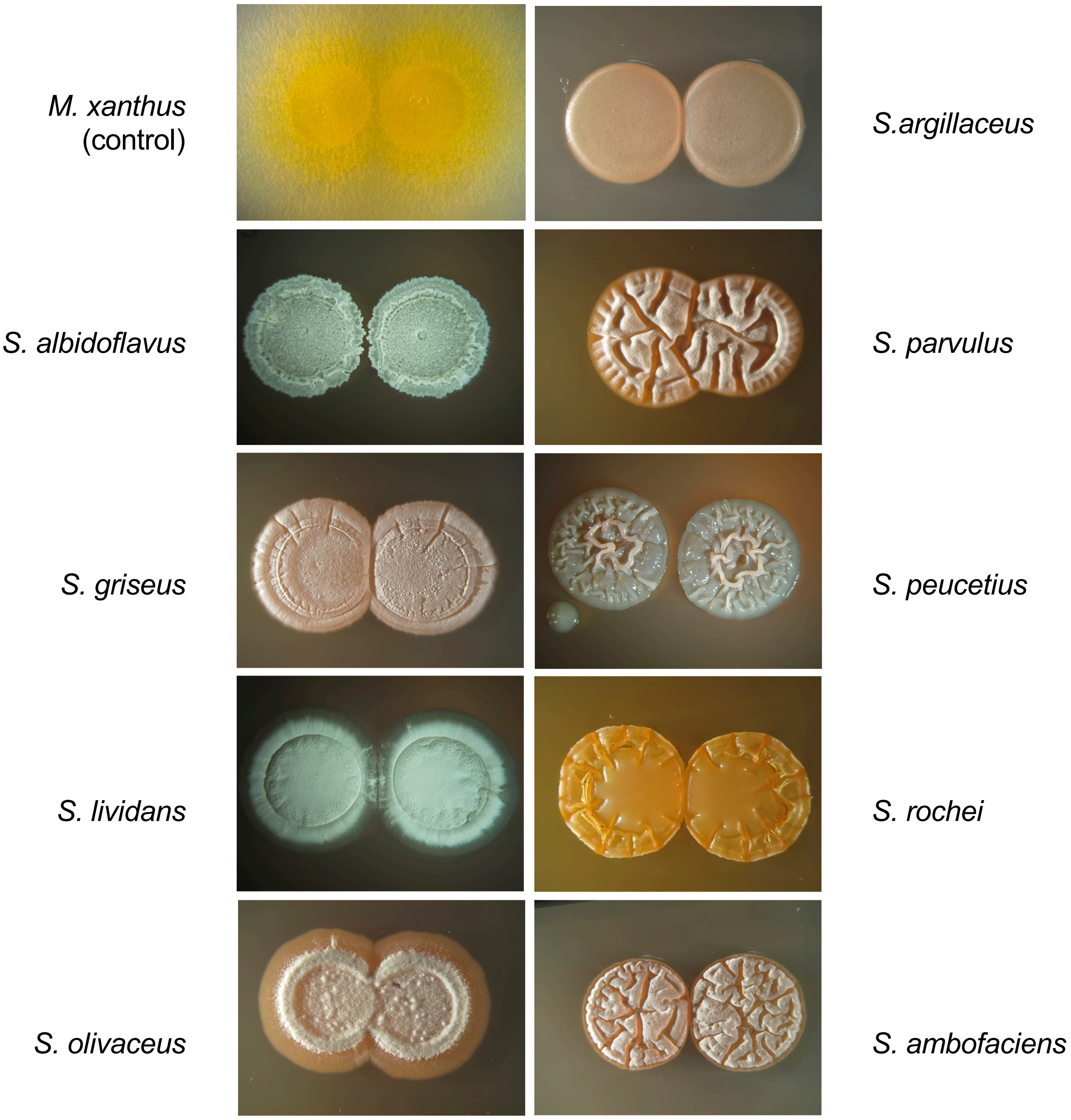

Supplementary Figure S1. Control spots of the different species used in Figure 1 on CYE plates: Two spots of each specie were used. The same order of the Figure 1 is followed. Pictures were taken from the top of the Petri dishes 7 days after the plates were inoculated.

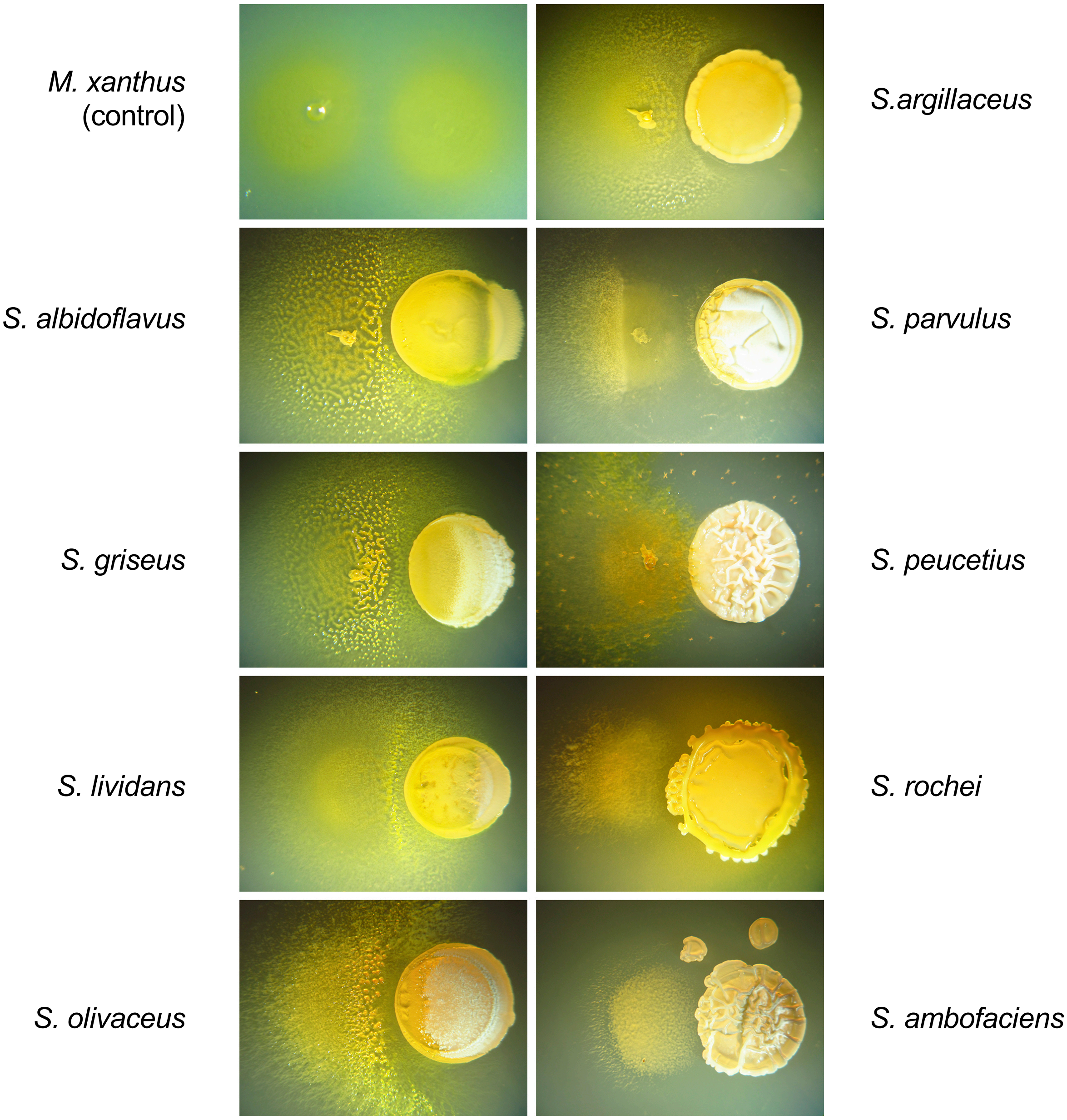

Supplementary Figure S2. Predatory activity of *M. xanthus* DK1622 on different *Streptomyces* species on CTT plates: A spot of *M. xanthus* is on the left of each photograph; the spot on the right corresponds to the different *Streptomyces* prey indicated. Two drops of *M. xanthus* were used as a control. Pictures were taken from the top of the Petri dishes 7 days after the plates were inoculated.

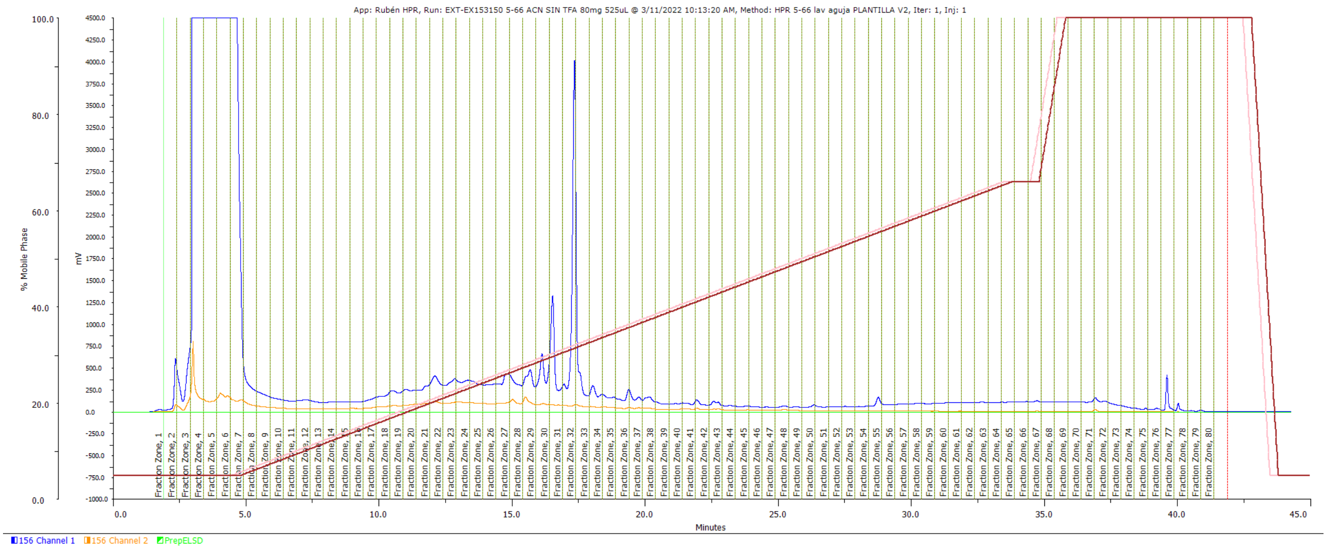

Supplementary Figure S3. Purification of the active components present in the *S. griseus* active E3 fraction. Preparative reversed-phase C-18 HPLC Agilent Zorbax profile at 210 nm (blue) and 280 nm (red) of the 3 kDa active eluent fractionation. Demethylenenocardamine and nocardamine were detected in fractions F30 and F31, corresponding to a well-resolved peak at 17.5 min.

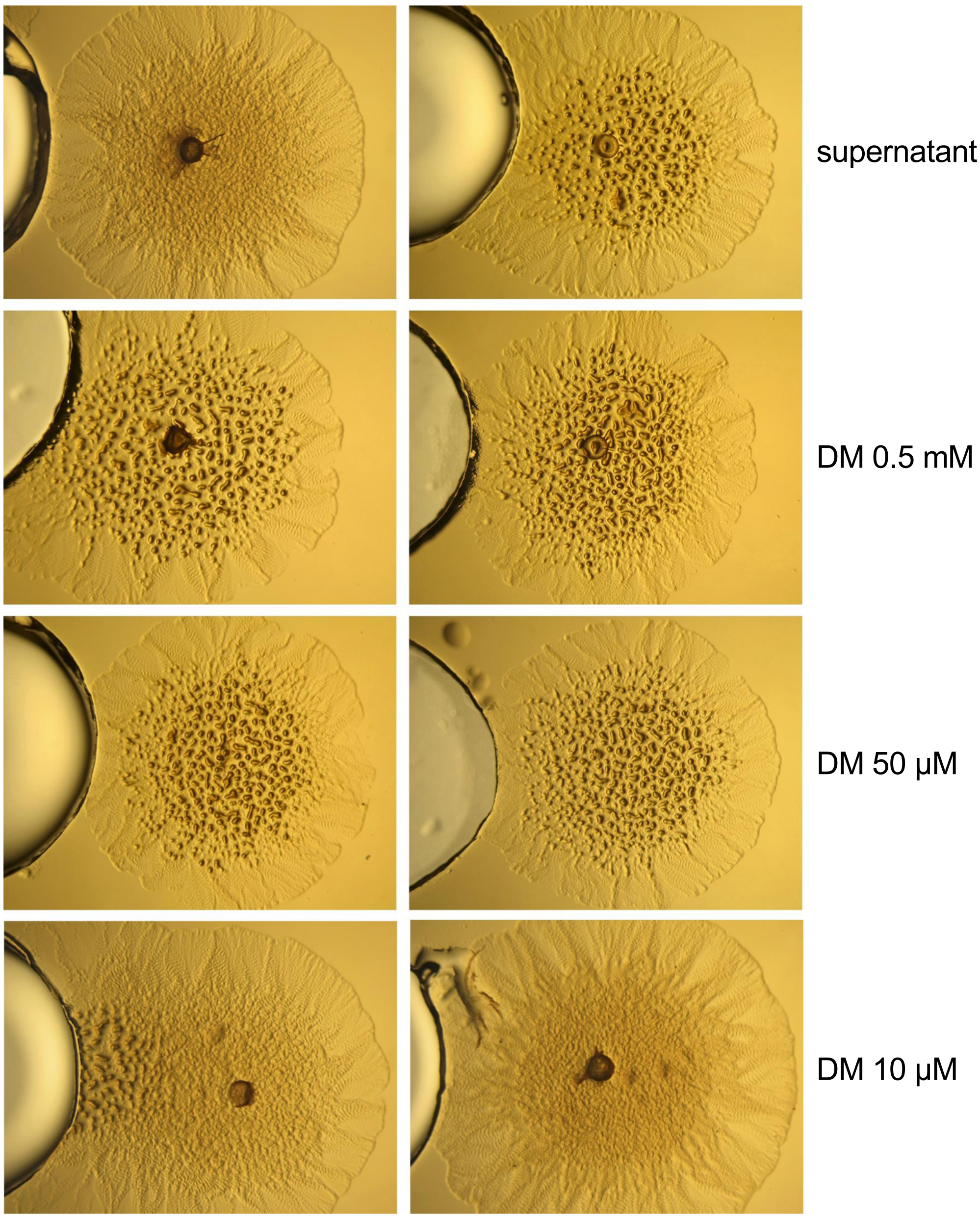

Supplementary Figure S4. Effect of the siderophore deferoxamine mesylate on *M. xanthus DK1622* in CYE. Effect on the behavior of *M. xanthus* (spot on the right of each photograph) of different amounts of deferoxamine mesylate (DM 10, 20, 50 µM; 0.1, 0.5, 1 mM) added to the well on the left of each *M. xanthus* spot. *S. griseus* supernatant was used as a positive control for aggregates formation and H_2O as a negative control.

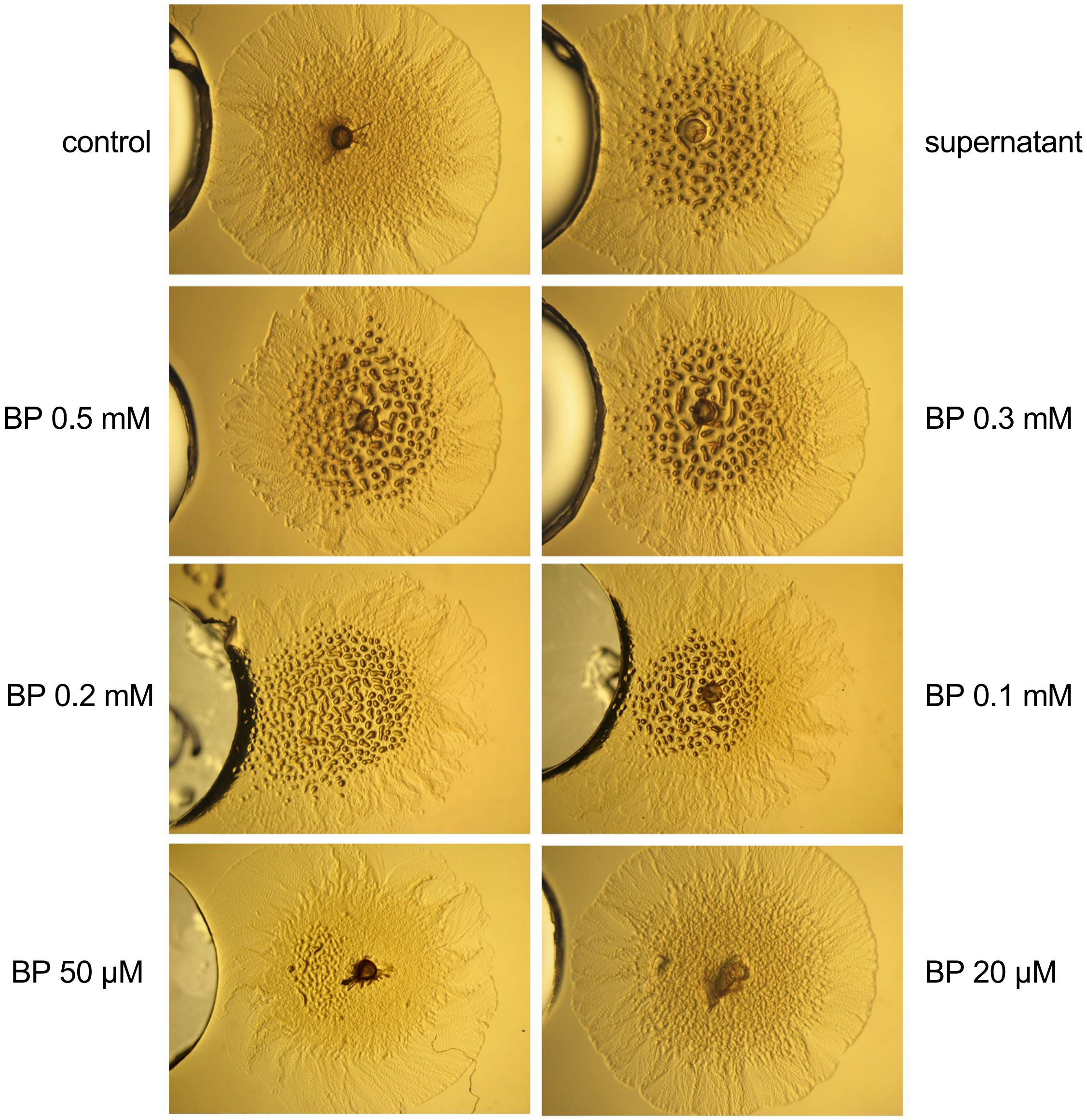

Supplementary Figure S5. Effect of the siderophore bipyridyl on *M. xanthus DK1622* in CYE. Effect on the behavior of *M. xanthus* (spot on the right of each photograph) of different amounts of bipyridyl (BP 20, 50 µM; 0.1, 0.2, 0.3, 0.5 mM) added to the well of the left of each spot of *M. xanthus*. *S. griseus* supernatant was used as a positive control for aggregates formation and H_2O as a negative control.

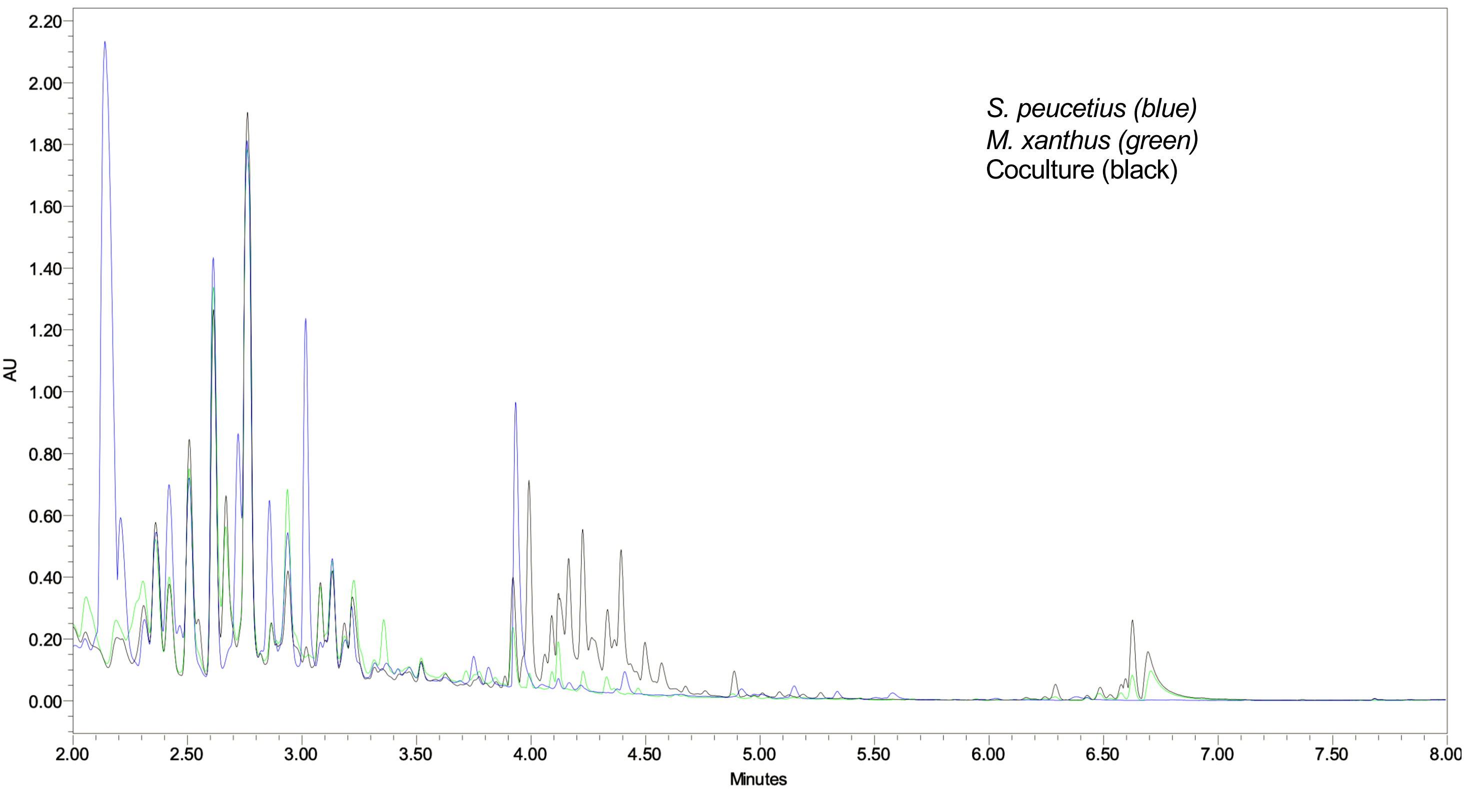

Supplementary Figure S6. Analysis of the compounds produced by the interaction of *M. xanthus DK1622 and S. peucetius* on solid CYE. UPLC analysis of the compounds produced by *S. peucetius* (blue), *M. xanthus* (green), and the co-culture of both microorganisms (black).

A

B

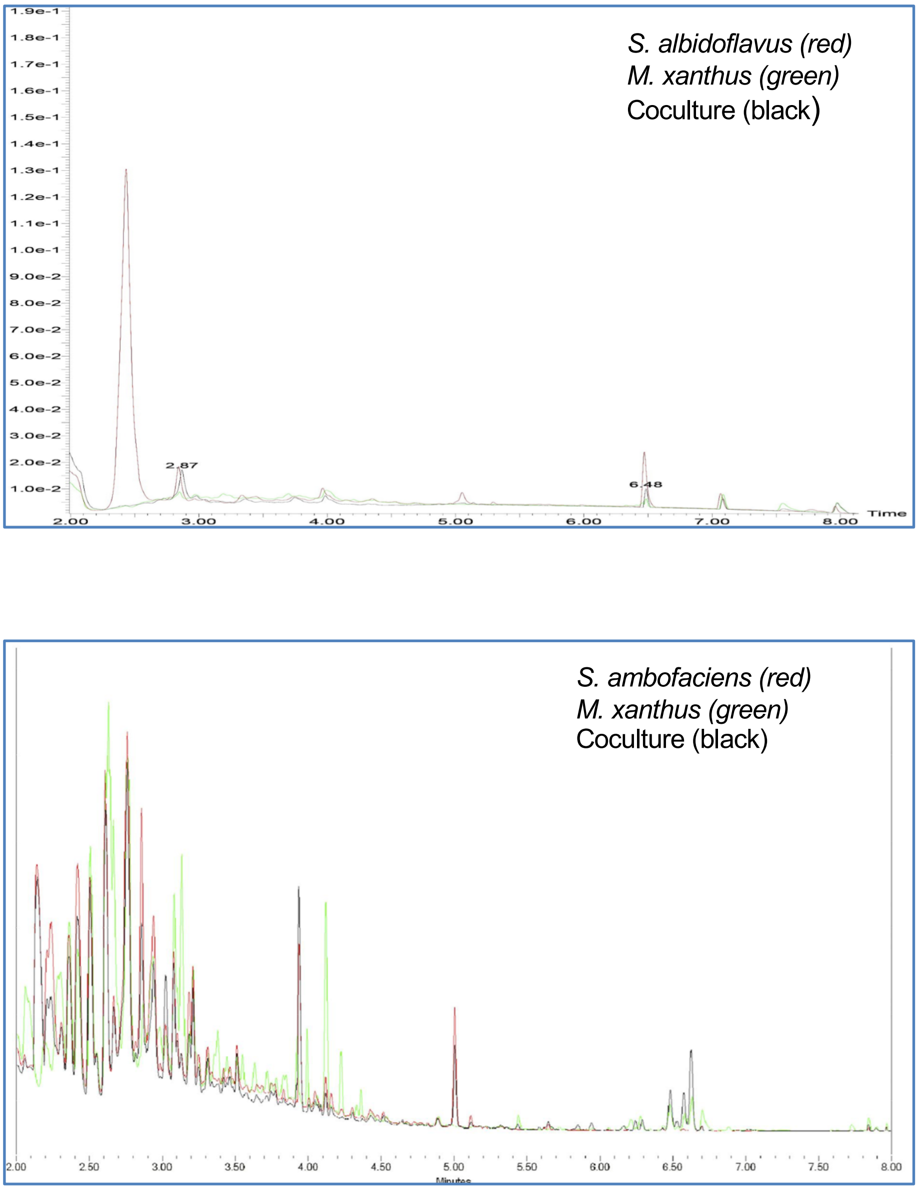

Supplementary Figure S7. Analysis of the compounds produced by the interaction of *M. xanthus DK1622 and S. albus* or *S. ambofaciens* on solid CYE. UPLC analysis of the compounds produced by A) *S. albus* (red), *M. xanthus* (green), and the co-culture of both microorganisms (black); B) *S. ambofaciens* (red), *M. xanthus* (green), and the co-culture of both microorganisms (black).

A

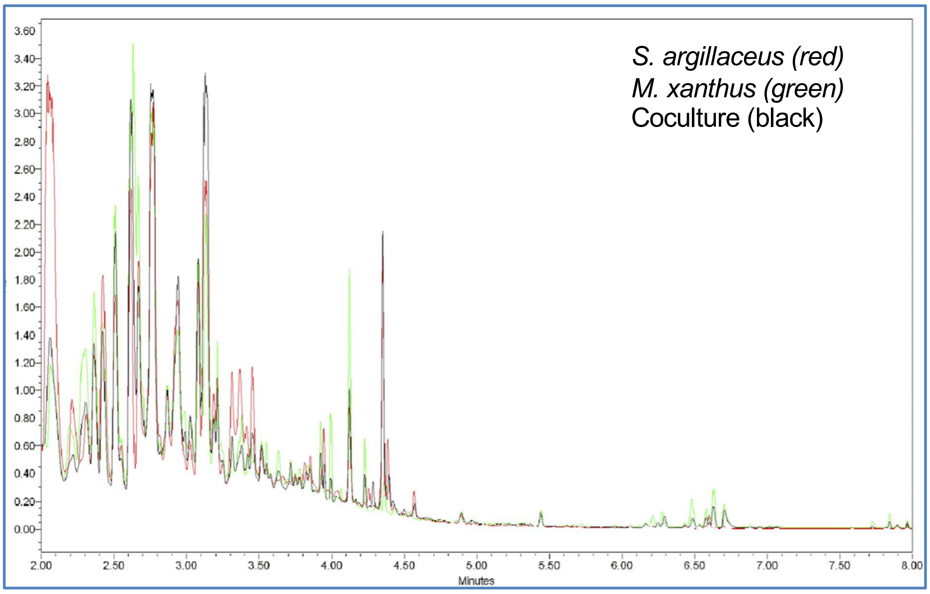

B

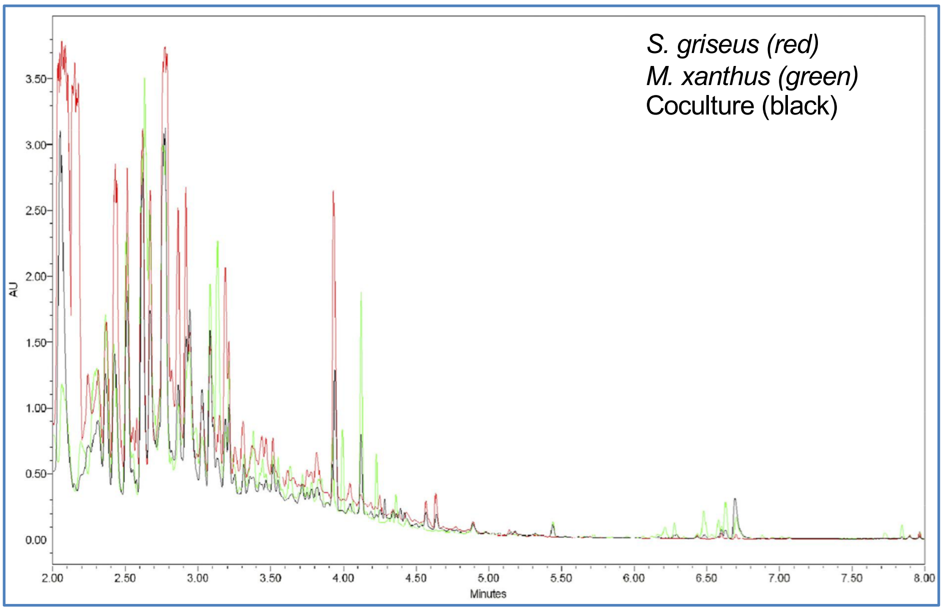

Supplementary Figure S8. Analysis of the compounds produced by the interaction of *M. xanthus DK1622 and S. argillaceus* or *S. griseus* on solid CYE. UPLC analysis of the compounds produced by A) *S. argillaceus* (red), *M. xanthus* (green), and the co-culture of both microorganisms (black); B) *S. griseus* (red), *M. xanthus* (green), and the co-culture of both microorganisms (black).

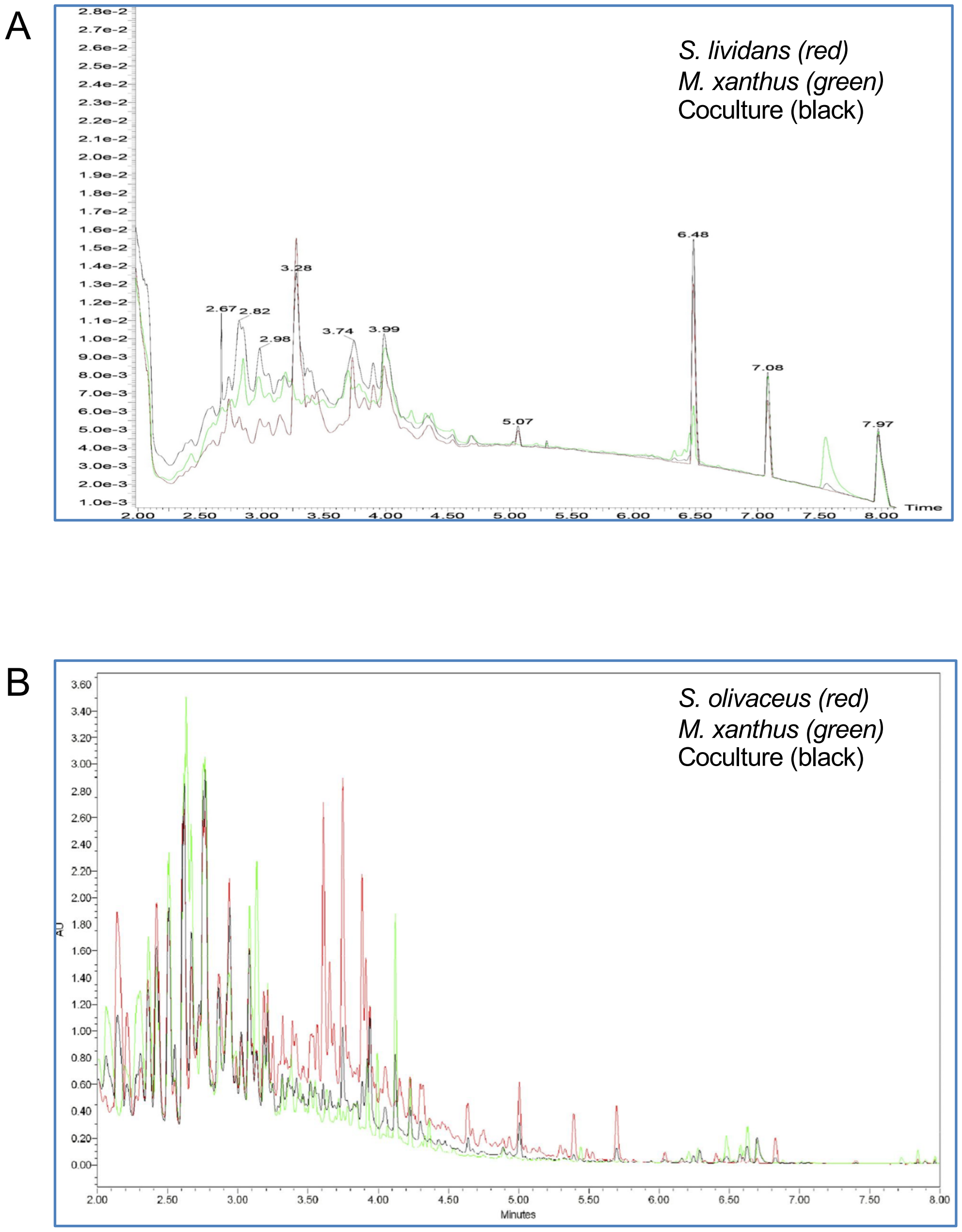

Supplementary Figure S9. Analysis of the compounds produced by the interaction of *M. xanthus DK1622 and S. lividans* or *S. olivaceus* on solid CYE. UPLC analysis of the compounds produced by A) *S. lividans* (red), *M. xanthus* (green), and the co-culture of both microorganisms (black); B) *S. olivaceus* (red), *M. xanthus* (green), and the co-culture of both microorganisms (black).

A

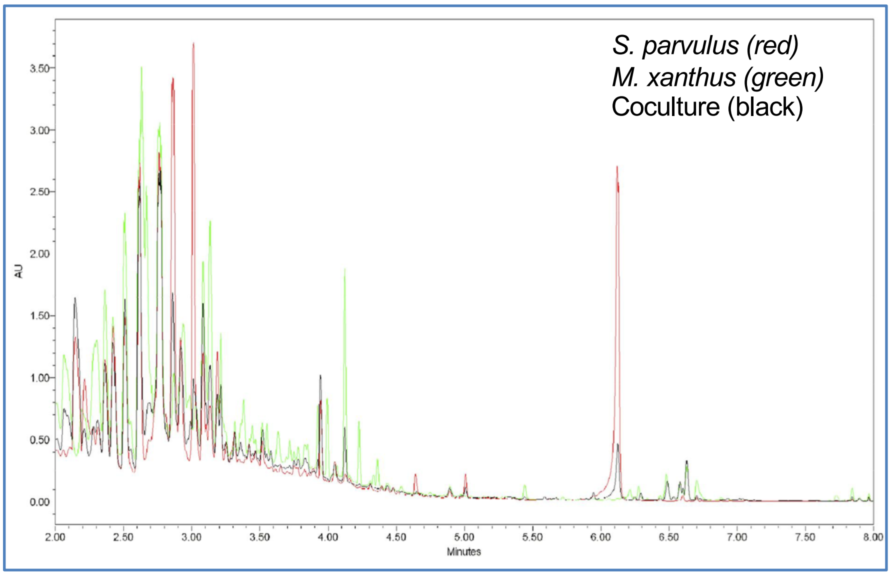

B

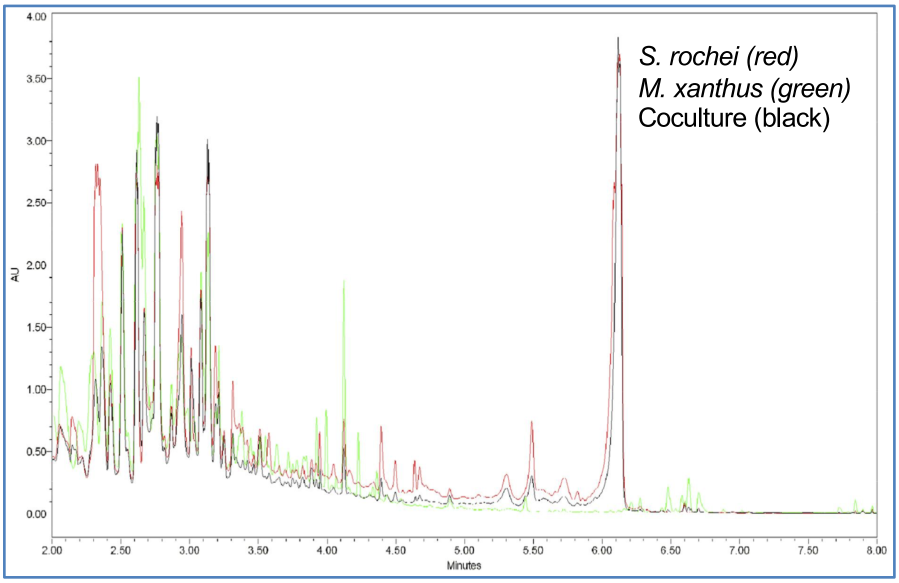

Supplementary Figure S10. Analysis of the compounds produced by the interaction of *M. xanthus DK1622 and S. parvulus* or *S. rochei* on solid CYE. UPLC analysis of the compounds produced by A) *S. parvulus* (red), *M. xanthus* (green), and the co-culture of both microorganisms (black); B) *S. rochei* (red), *M. xanthus* (green), and the co-culture of both microorganisms (black).

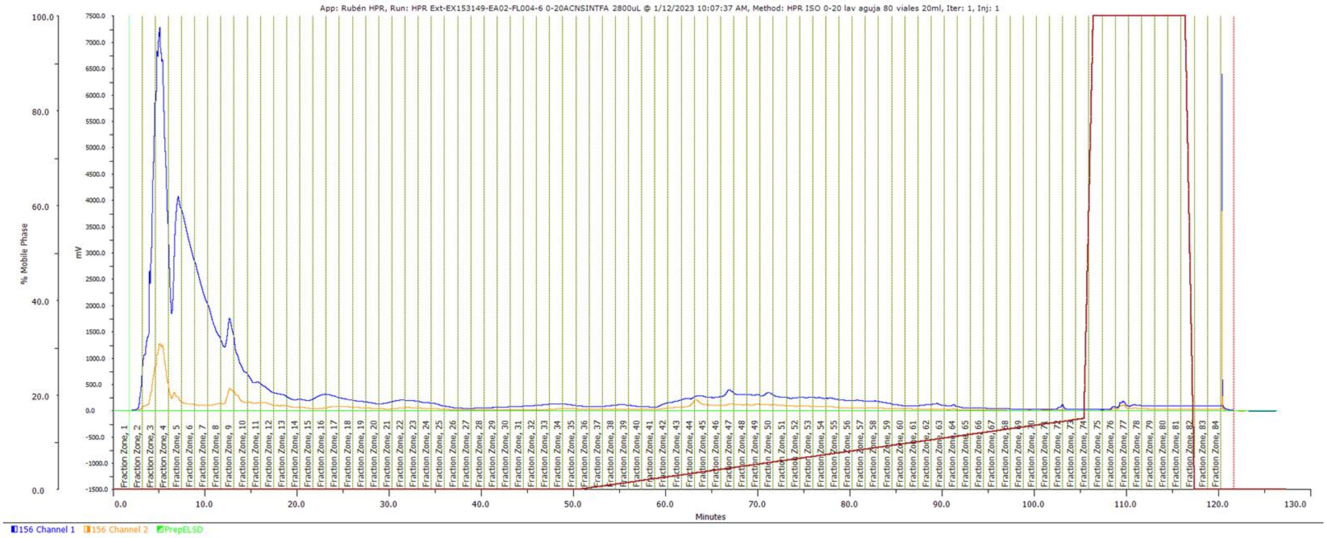

Supplementary Figure S11. Purification of the active components present in *S. peucetius* aqueous extract from active E3 fraction. Preparative reversed-phase C-18 HPLC Waters T3 profile at 210 nm (blue) and 280 nm (red) of the HP-20 aqueous non-retained extract fractionation of the 3 kDa active eluent. The activity was observed in fractions F13-F15 corresponding to two broad unresolved peaks at 20-21 min.
